# Adult neurogenesis and the microbiota-gut-brain axis in farm animals: underestimated and understudied parameters for improving welfare in livestock farming

**DOI:** 10.3389/fnins.2024.1493605

**Published:** 2024-11-27

**Authors:** Katharina M. Hillerer, Ulrike Gimsa

**Affiliations:** Research Institute for Farm Animal Biology (FBN), Dummerstorf, Mecklenburg-Vorpommern, Germany

**Keywords:** farm animals, neuroplasticity, animal welfare, environmental enrichment, microbiota-gut-brain axis, stress coping

## Abstract

Welfare in commercial livestock farming is becoming increasingly important in current agriculture research. Unfortunately, there is a lack of understanding about the neuronal mechanisms that underlie well-being on an individual level. Neuroplasticity in the hippocampus, the subventricular zone (SVZ), the olfactory bulb (OB) and the hypothalamus may be essential regulatory components in the context of farm animal behaviour and welfare that may be altered by providing environmental enrichment (EE). The importance of pre-and probiotics as a form of EE and the microbiota-gut-brain axis (MGBA) has come under the spotlight in the last 20 years, particularly in the contexts of research into stress and of stress resilience. However, it could also be an important regulatory system for animal welfare in livestock farming. This review aims to present a brief overview of the effects of EE on physiology and behaviour in farm animals and briefly discusses literature on behavioural flexibility, as well as inter-individual stress-coping styles and their relationship to animal welfare. Most importantly, we will summarise the literature on different forms of neural plasticity in farm animals, focusing on neurogenesis in various relevant brain regions. Furthermore, we will provide a brief outlook connecting these forms of neuroplasticity, stress, EE, the MGBA and welfare measures in modern livestock farming, concentrating on pigs.

## 1 Introduction

Commercial livestock farming typically raises animals in more controlled, relatively barren environments than their wild counterparts. Indeed, livestock animals show cognitive bias, self-control, and self-awareness, develop complex social relationships and are capable of learning (for review, see Doyle, 2017; Marino, 2017; Marino and Colvi, 2015; Nawroth et al., 2019). However, less complex housing conditions may limit their cognitive and emotional capacity due to compromised neurobiological development. Moreover, barren environments in livestock farming may contribute to such negative emotions and affective states as fear, anxiety and depression, as well as abnormal, undesirable behavioural patterns like stereotypies and injurious inter-individual interactions, which actually lead to reduced positive welfare states (Campbell and Lee, 2021; Crump et al., 2018; Lecorps et al., 2021; Mellor, 2015 for review). Improving animal housing conditions by increasing environmental and social complexity, also called environmental enrichment (EE), is a fundamental strategy to ensure that the animals are best adapted throughout their productive lives. Although research clearly shows a positive impact of EE across livestock species on such indirect measures as cognitive processing, affective states, emotions, judgement biases and stress response (Bolhuis et al., 2013; Grimberg-Henrici et al., 2016; Oesterwind et al., 2016 and see Okon-Singer et al., 2015 for review), which are not only intricately linked, but which have also been associated with measures of neuroplasticity. What underpins them neurobiologically remains largely understudied in farm animals. This is at least partly due to difficulties in analysing brain function in larger livestock species compared to rodents, as well as comparative novelty of applying neurobiological techniques in animal welfare research (Campbell and Lee, 2021 for review). Nevertheless, increasing our knowledge about the influence of housing conditions and EE on neuronal measures like neuroplasticity would be an essential step towards a better understanding of how to improve the welfare of individual livestock animals.

## 2 The importance of environmental enrichment for farm animal welfare

### 2.1 Environmental enrichment and its relation to stress-coping and behavioural flexibility

**Figure 1:**
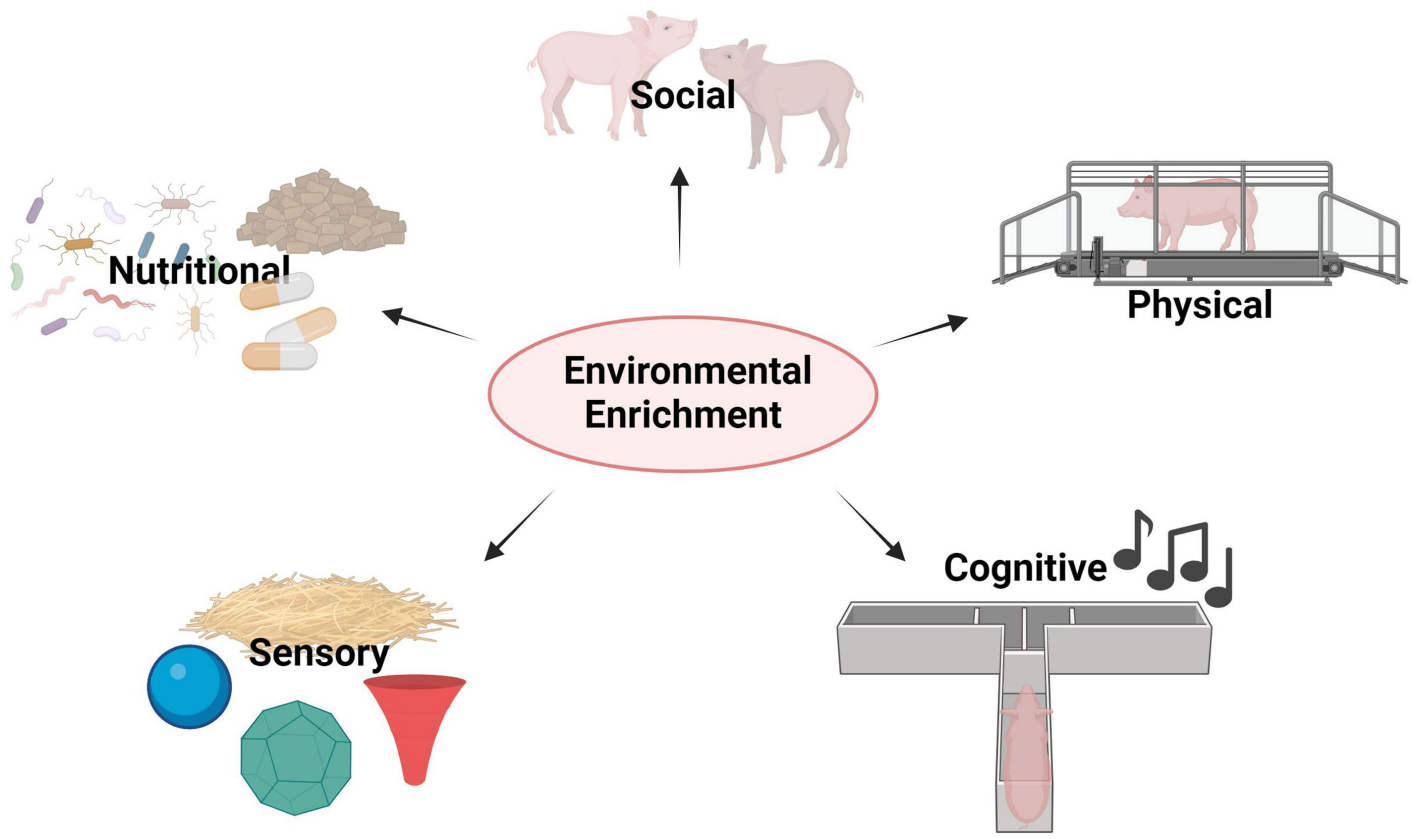
Graphical summary of EE types in farm animals; EE may be provided by the implementation of (1) Sensory tools (for example straw bedding, plastic balls, deformable and digestible material in various shapes), (2) Nutritional tools (e.g., by using pre/probiotics or specifically enriched chow), (3) Social tools (such as keeping animals in stable social groups), (4) Physical tools (for instance, the opportunity for access to outside with sufficient space to move freely and the opportunity to run about) and (5) Cognitive tools (for example daily music exposure, cognitive challenges in the form of discrimination/operant/puzzle box tasks).

Animal welfare is an increasingly important issue in current agricultural research, due to the growing consumer demand and intensification of livestock production, which simultaneously contributes to concerns about the health and well-being of animals bred for their products (Mellor, 2015; Nawroth et al., 2019, for review). Animal welfare is characterised as the result of physical and psychological well-being (for review, see Coria-Avila et al., 2022; Duncan, 1987) and is expected to occur when animals are free from hunger, thirst, malnutrition, discomfort, stress, fear, and pain or if animals express their natural behavioural repertoire (Coria-Avila et al., 2022, for review). The concept of animal welfare is based on research findings that animals have subjective emotions and can feel pleasure and suffering (Nawroth et al., 2019, for review). Importantly, welfare is not a constant state but rather the result of specific brain dynamics that render motivated behaviours. Impaired welfare can be expected when the allostatic load from repeated, chronic, unpredictable and/or uncontrollable conditions compromises the physiological and psychological basis for health due to an impaired allostatic adjustment (for a review, see Korte et al., 2007; Mcewen, 1998; McEwen and Stellar, 1993). Many physiological and behavioural adjustments labelled as stress responses may rather be seen as adaptive changes, through which the animal responds to predictable/unpredictable events (Koolhaas et al., 2011; Romero et al., 2009, for review), and these adjustments are also dependent on individual coping styles (de Boer et al., 2017, for review). Individual stress-coping abilities and variations in stress responsiveness are common phenomena, seen not only in laboratory animals (Bosch et al., 2006; Castanheira et al., 2017; Neumann et al., 2005; Silva et al., 2010 and see Koolhaas et al., 1999; Veenema and Neumann, 2007, for review), but also in livestock. More specifically, different stress-coping styles in pigs, which can be defined as sets of behavioural and physiological responses to stress, have been characterised as reactive vs. proactive. Reactive pigs show a high post-stress HPA-axis activation and a robust increase in cortisol (CORT) levels, low sympathetic adrenal-medullary system activity, low levels of aggression, low risk-taking, and high behavioural flexibility as measured by the Open-Field/Novel-Object test. In contrast, proactive pigs are characterised by low CORT responses, high sympathetic activity, aggressive-, as well as rigid and high-risk behaviour (Kanitz et al., 2019). Inter-individual variations in responsiveness can be of particular importance in animal welfare since certain personality traits may enhance or inhibit stress-coping abilities (Griffin et al., 2015, for review). Thus, a changing or challenging environment may not affect some animals while negatively impacting others. It has generally been accepted that one of the main characteristics of contrasting coping styles is behavioural flexibility (Coppens et al., 2010, for review), which may be measured by tests of cognitive functions and influenced by chronic stress. Indeed, cognition is inextricably linked with stress coping, emotions and thus mental health, as seen in human studies (Barnett et al., 2012; Karestan et al., 2009 and see Burger, 2010 and Martin et al., 2007 for review). Evidence from human literature to farm animals shows that individuals lacking environmental complexity or enrichment may be more likely to show (1) negative affective states such as aggression, (2) increased susceptibility to negative emotions, (3) reduced ability to experience positive states, (4) lower emotional intelligence, (5) reduced stress-coping abilities and (6) reduced behavioural flexibility (Campbell and Lee, 2021, for review). Despite the knowledge of these consequences, commercial indoor animal husbandry systems often offer minimal environmental stimulation and a lack of opportunity for farm animals to express their inherent behaviours. Barren slatted concrete floors with no rooting material preclude the expression of natural behaviours like those exhibited under normal environmental conditions (Van de Weerd and Day, 2009), such as rooting, wallowing and exploring, particularly in pigs (Studnitz and Pederson, 2007), thus leading to harmful social and aggressive behaviour towards conspecifics (Fraser et al., 1991). Indeed, there is growing interest in implementing EE for farm animals in intensive production systems to improve animal welfare while maintaining high productivity (Beattie et al., 1996; Fraser et al., 1991; Jansen et al., 2009; Zebunke et al., 2013). Although there is no standardised form of EE, it usually comprises a physical, sensory, social, cognitive or nutritional component (see Figure 1) (Newberry and Wood-Gush, 1988; Van de Weerd et al., 2003) to increase the number and range of normal behaviours and the ability to deal with behavioural and/or physiological challenges, while preventing abnormal behaviours. In choosing the right form of EE, it must be borne in mind that EE preferences are not only species-specific, but also sex-specific (Van de Weerd et al., 2003; Wurbel, 2007). Unfortunately, there is a lack of studies evaluating the effect on each sex (Simpson and Kelly, 2011, for review).

There seems to be a notable time-dependent factor, with EE being particularly effective during sensitive developmental windows of brain development, i.e., during the early prenatal and postnatal period as seen in rodents (Brummelte et al., 2007; Cymerblit-Sabba et al., 2013; MacRae et al., 2015). Consequently, EE has been shown to buffer the adverse effects of parental neglect and early-life stress at the time of weaning, whereas no effect was observed when EE was applied in later life (Bredy et al., 2003). Specifically, EE offspring showed better behavioural adaptability in response to novel contexts and elevated levels of GLUR1 and GABA in the PFC, indicative of a better stress regulatory system (Connors et al., 2015). Interestingly, the benefits of EE seem to be mediated by changes in maternal care (Connors et al., 2015), which is known to affect not only the physiology but also behaviour of the offspring (Francis et al., 1999a,b; Francis and Meaney, 1999).

The results from rodent studies could be particularly interesting in modern animal husbandry, where early weaning represents a stressor for the offspring. Putting the results from rodent studies in the context of livestock farming suggests that applying EE is particularly important during the prenatal phase to minimise potential behavioural disturbances in the offspring during their animal husbandry lives.

### 2.2 Behavioural and hormonal measures to assess the effect of environmental enrichment

Although studies do reveal EE’s beneficial effects on outcome such as behaviour, stress response, performance and productivity (Mkwanazi et al., 2019, for review), the measurement of welfare and stress resilience in livestock animals is challenging. There are only a few valid scientific measures for reliably assessing their effects (Rault et al., 2018). Nevertheless, the positive effects of EE have been shown on physiological parameters like CORT levels through the minimisation of stress levels and promotion of inherent pig behaviour in intensive production systems. As a result, pigs reared in barren environments without any substrates show higher CORT concentrations than pigs in an enriched environment (de Jong et al., 2000; Pearce and Paterson, 1993; Peeters et al., 2006; Van de Weerd and Day, 2009; Yonezawa et al., 2012). One important factor to consider when assessing CORT levels in the context of stress is its diurnal regulation. Consequently, assessing the effect of EE on the circadian rhythm of CORT plasma levels may be a more reliable measure of outcomes than single CORT measurements (Geverink et al., 2002; Ruis et al., 1997). Bypassing this problem, measuring hair CORT may be a reasonable alternative, for one thing it is non-invasive and, for another, rather independent of the fluctuations seen in plasma levels (see Heimbürge et al., 2019, for review). Aside from changes in CORT levels, the body temperature, growth performance and meat quality have been demonstrably altered in pigs without EE (Mkwanazi et al., 2019, for review).

EE's positive effects on pig behaviour are well studied and numerous (Mkwanazi et al., 2019, for review). Aggression among pigs is a common and normal behaviour primarily driven by forming a social/dominance hierarchy (Turner and Tilbrook, 2006, for review). However, the high levels of aggression often seen in uncomfortable environments in barren housing systems can further exacerbate existing high levels of aggression, constituting a challenge to welfare in pig livestock farming (Bolhuis et al., 2005; Turner and Tilbrook, 2006). Another sign that the environment of intense farming fails to meet pigs’ behavioural needs is the occurrence of harmful social behaviour (e.g., tail-biting) (Beattie et al., 2000; van de Weerd et al., 2005). Importantly, EE has been shown to successfully reduce both the level of aggressive interaction among pigs and harmful social behaviours by increasing the time spent on the substrates provided (Beattie et al., 2000). Although play behaviour is not typical in adult pigs, research suggests that pigs in EE exhibit more play behaviour, which may increase behavioural flexibility and, thus, welfare in the long term (Bolhuis et al., 2005; Kelly et al., 2000; Newberry and Wood-Gush, 1988; Spinka et al., 2001).

### 2.3 Neurobiological mechanisms underlying the effects of environmental enrichment

Although revealing ultimate and proximate mechanisms behind these analogous behaviours and investigating causes and consequences of personality-cognitive covariation is a growing field of research in domesticated animals (Goursot et al., 2019; Kanitz et al., 2019 and see Finkemeier et al., 2018; Griffin et al., 2015; Sih, 2012, for review), the underlying neuronal mechanisms that drive these variations are largely unknown and underestimated. Neural plasticity is one parameter that could be a particularly interesting biomarker in the context of stress coping styles, behavioural flexibility, emotional intelligence and thus individual welfare, given the fact that there seems to be a correlation between measures of neural plasticity, in other words, adult neurogenesis, EE/complexity, cognition, stress and vice versa (Overli and Sorensen, 2016, for review). Therefore, examining adult neurogenesis in different relevant brain regions, such as the subgranular zone (SGZ) of the hippocampus, the subventricular zone (SVZ), the olfactory bulb (OB) and the hypothalamus may be a promising avenue for understanding differences in individual stress coping and how EE may lead to improved livestock welfare at an individual level.

The following sections summarise the literature available on neuroplasticity in specific brain regions (i.e., SGZ, SVZ, OB and hypothalamus) of farm animals (i.e., pigs, cattle, goats and sheep) and aim to draw correlations with behavioural measures like cognition and how these measures may be linked to welfare in animal husbandry.

## 3 Neuroplasticity in farm animals: state of the art and comparison with other mammals

### 3.1 Neurogenic zones and specific markers for neurogenesis in farm animals

Whereas there are significant anatomical differences between rodent and human brains, the brains of farm animals such as pigs, cattle, goats and sheep share some critical structural and functional characteristics (Abbott, 2014). Specifically, they have a similar pattern of brain development with rapid growth during the perinatal period, a highly evolved gyrencephalic neocortex, a comparable cortical organisation and anatomical structure, as well as a similar grey/white matter ratio and regional distribution of neurotransmitter systems (Conrad et al., 2012; Henry et al., 1996; Ishibashi et al., 2012; Pond et al., 2000; Quinones-Hinojosa et al., 2006; Rakic, 2009; Ryan et al., 2018; Zhang and Sejnowski, 2000 and see Dobbing and Sands, 1979; Quinones-Hinojosa et al., 2007, for review). Thus, although somewhat less documented and understood, it is not surprising that adult neurogenesis as seen in tree shrews, marmosets, macaques and humans (Cameron et al., 1993b; Eriksson et al., 1998; Gould et al., 1997; Gould et al., 1999b; Kempermann et al., 1997a), has also been observed in farm animals (Brus et al., 2010; Corona et al., 2017; Dhari et al., 2021; Guidi et al., 2011; Huguet et al., 2023; Levy et al., 2019; Rodriguez-Perez et al., 2003).

In the adult mammalian brain, two neurogenic regions are generally accepted: the SVZ of the lateral ventricle and the SGZ of the hippocampus (Altman and Das, 1965; Brazel et al., 2003; Palmer et al., 1995; Reynolds and Weiss, 1992; Sanai et al., 2004). Multipotent neural stem/progenitor cells (NSCs), also known as precursor cells, which reside in the anterior portion of the SVZ in the walls of the lateral ventricles, migrate along the rostral migratory stream (RMS) into the OB, where they differentiate into neurons (Okano, 2002, for review). Although reports are conflicting, the production of new neurons also seems to occur in the neocortex, the amygdala and the hypothalamus (Dayer et al., 2005; Fowler et al., 2002). At least the latter seems to play an important functional role in sheep, where it regulates seasonal plasticity in the context of reproductive function (Batailler et al., 2016; Kokoeva et al., 2005; Xu et al., 2005, for review see Lee and Blackshaw, 2012 and Migaud et al., 2010).

**Figure 2:**
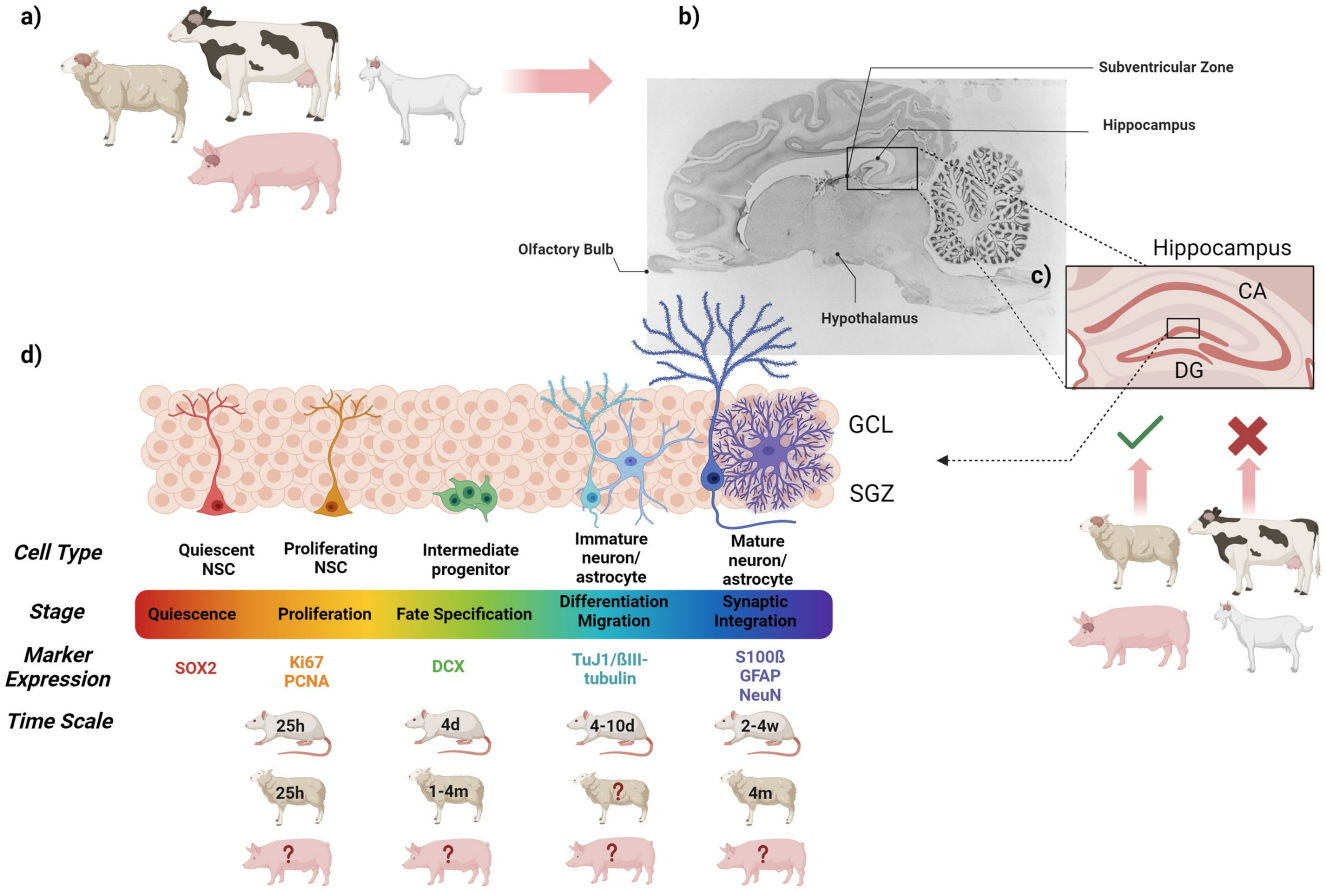
Graphical summary showing **(a)** the farm animals discussed in this review, **(b)** the brain regions known for their neurogenic potential and so far studied in these farm animals (sagittal section of the pig brain adapted from (Felix et al., 1999), **(c)** magnification of the mammalian hippocampus including the DG and CA (cornu ammonis) region, which has been investigated in sheep and pigs, but not yet in cattle and goats, **(d)** the dynamics of adult hippocampal neurogenesis, including specific cell types, stages of hippocampal neurogenesis, markers used to investigate hippocampal neurogenesis in farm animals and approximate times for the different stages in rodents, sheep and pig (where known). CA, Cornu Ammonis; d, Days; DG, Dentate Gyrus; GCL, Granule cell layer; m, Month; NSC, Nuclear Stem Cell; SGZ, Subgranular zone; w, Weeks.

Adult hippocampal neurogenesis, which occurs in the neurogenic region of the DG, the SGZ, comprises at least four distinct processes: cell proliferation, differentiation, migration and cell survival (see also Figure 2). The SGZ harbours a resident population of cells believed to be multipotent NSCs (Kempermann et al., 2004). NSCs have some characteristics of astroglial cells and, as such, express the astrocytic marker glial fibrillary acid protein (GFAP)—however, they are not themselves glial cells. They are either in a quiescent stage, in which they are in G_0_ of the cell cycle (when they express SOX2, a transcription factor essential for the maintaining self-renewal of stem cells), or self-renewing via mitosis (when they express proliferating cell nuclear antigen (PCNA)), or the proliferative marker Ki67. NSCs that are actively engaged in cycling, give rise to daughter cells (progenitor cells), which act as transiently amplifying cells (Mckay, 1997; Seaberg and van der Kooy, 2002) and express the microtubule-associated protein doublecortin (DCX), a marker for commitment to a neuronal phenotype (Brown et al., 2003; Seri et al., 2004; Seri et al., 2001). These progenitor cells re-enter the cell cycle to divide again, producing daughter cells that become post-mitotic and differentiate. In rodents, the majority of these cells (~70%) (Cameron et al., 1993a) differentiate into neurons (Cameron and McKay, 2001), as they express the neural nuclei protein NeuN (Kempermann, 2006). In contrast, only a few (~10%) (Cameron et al., 1993a) become astroglial cells that express GFAP (Kempermann et al., 1997b). After differentiation, cells migrate to the granule cell layer (GCL), where, after approximately 7 weeks following division, the new cells are functionally indistinguishable from older cells (Zhao and Overstreet-Wadiche, 2008) and are electro-physiologically integrated into the circuitry (Hastings and Gould, 1999; Stanfield and Trice, 1988; van Praag et al., 2002). However, differences in lifespan and brain expansion accompanied by gyrification and topographical complexity lead to dramatic differences in the dynamics, cellular composition and morphological organisation of adult neurogenesis in livestock animals (Levy et al., 2017, for review). Thus, generation, migration and differentiation are much longer in primates and farm animals than in rodents (for more details, see Section 3.2). SVZ-derived neural stem and progenitor cells proliferate as multipotent neurospheres, expressing the neural progenitor and neurodevelopmental markers SOX2, GFAP, DCX and Ki67 (Ara and De Montpellier, 2013). Indeed, NSCs from the SVZ have been shown to produce neurons and glia *in vivo* and *in vitro* (Guidi et al., 2011; Liard et al., 2009; Yin et al., 2011) and to migrate to the hippocampus, where they become incorporated in the hippocampal circuitry (Nakatomi et al., 2002). The fact that DCX+ cells with morphologies of migrating neuroblasts are additionally found in the striatum, the prefrontal cortex and the anterior cingulate cortex (Aoyagi et al., 2018; Lois et al., 1996; Morton et al., 2017; Sanai et al., 2011 and see Lazarini and Lledo, 2011, for review) suggests that SVZ NSCs are the main source of new neurons in the neocortex, contributing to adult gyrencephalic growth by grey matter maturation, at least in humans and pigs (Ara and De Montpellier, 2013; Morton et al., 2017; Paredes et al., 2016; Sanai et al., 2005). In species with strong olfaction, such as rodents, sheep and pigs, NSCs located on the wall of the lateral ventricles function as primary precursors in the SVZ to continuously generate large numbers of interneurons, which radially migrate through the RMS to the OB, where they mature and mostly replenish granular GABAergic interneurons throughout life (Costine et al., 2015 and Gage and Temple, 2013; Ming and Song, 2011, for review).

As mentioned above, there is also evidence for a neurogenic niche in the hypothalamus. There, resident stem cells with the capacity to self-renew have been observed across numerous mammalian species, including mice (Kokoeva et al., 2007), rats (Pencea et al., 2001a; Xu et al., 2005), voles (Fowler et al., 2005), hamsters (Huang et al., 1998; Mohr and Sisk, 2013) and sheep (Batailler et al., 2014; Migaud et al., 2011; Migaud et al., 2010). However, the level of constitutive neurogenesis is lower than those seen in the SGZ and SVZ (Lee and Blackshaw, 2012, for review). Although the identity and location of hypothalamic NSCs are still unclear or at least debatable, as there has been no proper characterisation to date, specialised hypothalamic glial cells-tanycytes-may be the key cell type in this context (Haan et al., 2013; Robins et al., 2013). Indeed, they show characteristics of neural stem/progenitor cells by expressing the specific markers nestin, vimentin and SOX2 in all mammalian species studies conducted so far (Baroncini et al., 2007; Batailler et al., 2014; Bennett et al., 2009; Bolborea and Dale, 2013; Li et al., 2012; Rodriguez et al., 2005; Wei et al., 2002). Located along the border of the third ventricle, their morphology is similar to radial glia, and they exhibit long processes that project to the hypothalamic parenchyma to reach the fenestrated capillary network. As such, they are at the focus of central physiological regulation and may be particularly important in seasonal species, where the hypothalamus plays a critical role in the availability of thyroid hormones, which are mandatory for an appropriate seasonal alteration between breeding and non-breeding seasons (Nicholls et al., 1988).

### 3.2 Hippocampal neurogenesis in farm animals

To date, adult hippocampal neurogenesis has been shown in the neonatal, new-born and developing piglet brain, in prepubertal and adult pigs, as well as in various breeds of lambs and adult sheep and in different physiological, pathophysiological and behavioural contexts (Brus et al., 2010; Brus et al., 2013; Brus et al., 2014; Conrad et al., 2015; Hawken et al., 2009; Huguet et al., 2023; Swanson et al., 2017; Val-Laillet et al., 2017; Val-Laillet et al., 2018; Val-Laillet et al., 2020) (see also Table 1), whereas there are no reports about neural plasticity in the bovine or caprine hippocampus. So far, research in pigs has mainly investigated the outcome of diet (Huguet et al., 2023; Val-Laillet et al., 2017; Val-Laillet et al., 2020) or pathological interventions and conditions such as traumatic brain injury (Kinder et al., 2019), or infection (Conrad et al., 2015) on the neurogenic niche in the hippocampus. In sheep, most studies have focused on the interplay between the social-hormonal environment and neurogenesis in the DG. Given that stress, EE, cognition and welfare are closely linked, it is striking that no studies have addressed this issue up to now.

**Table 1:** The studies on adult neurogenesis in farm animals in the different neurogenic regions, i.e., the hippocampus, SVZ, OB and hypothalamus.

Brain region	Species (breed)	Sex	Age	Parameters (outcome)	Marker	Context/condition	Ref
HIPP	Pig (Göttingen minipig)	F/M	Juvenile (6–7 weeks) Adult	Proliferation	BrdU	Descriptive	Guidi et al. (2011)
	Pig (Landrace)	F	Prepubertal (19 weeks)	Immature neurons (↑ with supplementation)	DCX	Diet (probiotics, omega-3-fatty acid)	Huguet et al. (2023)
	Pig (Yucatan minipigs)	F/M	Juvenile (3 months)	Proliferation (↑ with supplementation) Immature neurons (↓ with supplementation)	Ki67, DCX	Diet (western diet during pregnancy)	Val-Laillet et al. (2017)
	Pig (Landrace)	F	Juvenile (3.5 m)	Hippocampal GCL volume (↑) Proliferation (↑ with supplementation) Immature neurons (↑ with supplementation)	Ki67, DCX	Diet (sodium butyrate)	Val-Laillet et al. (2018)
	Pig (Crossbred)	F/M	Neonatal (28 days)	Survival (↓ in M; ↔ in F after infection)	BrdU	Infection (respiratory viral infection)	Conrad et al. (2015)
	Pig (Landrace)	M	Juvenile (6 weeks)	NeuN cell numbers (↓ after TBI)	NeuN	TBI	Kinder et al. (2019)
	Sheep (Romney/Suffolk)	F	Adult young (3 years)	Short/Long-term survival (↔)	BrdU, PCNA	Descriptive	Low et al. (2013)
			Adult old (5–6 years)	Proliferation (↔)			
	Sheep (Île-de-France)	F	Adult (1–2 years)	Evidence for different cell types in DG Proliferation (↓)	BrdU, Ki67, DCX, GFAP	Descriptive/Parturition/Lactation	Brus et al. (2010)
	Sheep (Île-de-France)	F	Adult (2–3 years)	Stem progenitor (↔) Neuroblasts (↔) Neuronal differentiation (↑ with time since BrdU) Glial cells (↔)	BrdU, DCX, NeuN, SOX2, S100ß	Parturition (1, 2, 3, 4, and 8 m post BrdU)	Brus et al. (2013)
	Sheep (Île-de-France)	F	Adult (1.5-2y)	Proliferation (↓ with parturition) Neuroblasts (↔) Neuronal differentiation (↓with parturition) Survival (↔)	BrdU, Ki67, DCX, NeuN	Lactation/Interaction with young	Brus et al. (2014)
	Sheep (Île-de-France)	F	Adult (4–5 years)	Proliferation (↔) Neuroblasts (↔)	Ki67, DCX	Effect of OXT	Levy et al. (2019)
	Sheep (Romney)	M	Juvenile (6 weeks)	Proliferation, Differentiation (↔)	BrdU, PCNA, GFAP, NeuN	Exercise (Running)	Swanson et al. (2017)
SVZ	Pig (Yorkshire/Landrace)	F/M	Neonatal (0 day, 16 days, 42 days)	No. of DCX Clusters (↑ with age) Proliferation (Ki67 ↓ with BrdU post-inj. time)	BrdU, Ki67, DCX	Descriptive	Porter et al. (2022)
	Pig (n.a.)	F	Cell culture (1 day old brains)	Characterisation of NSCs	DCX, Ki67,GFAP, NeuN, TuJ1/ßIII-tubulin, SOX2	Descriptive	Ara et al. (2010)
	Pig (Yorkshire)	F/M	Neonatal/Juvenile (7 days, 1 month, 4 months)	Immature neurons (↑ with lesion)	DCX	TBI	Costine et al. (2015)
	Pig (Landrace)	M	Juvenile (6 weeks)	Immature neurons (↑ with lesion)	DCX	TBI	Kinder et al. (2019)
	Pig (n.a.)	F	Cell culture (2 days, 4 days, 8 days old brains)	Proliferation (↑ with Hypoxic Preconditioning)	BrdU	Hypoxic preconditioning	Ara and De Montpellier (2013)
	Pig (Yorkshire)	F	Neonatal/Juvenile (7 days, 7 weeks, 15 weeks)	Stem progenitor cell No. and proliferation (↓ with hypoxic treatment)	Ki67, SOX2	Congenital heart disease/Hypoxia	Morton et al. (2017)
	Pig (Yorkshire)	n.a.		Proliferation (↑ with CPB) Neuroblasts (↔)	Ki67, DCX, GFAP	CPB	Maeda et al. (2020)
	Pig (Yorkshire)	F	Neonatal/Juvenile (3–4 weeks)	Stem progenitor cells, neuroblasts, Proliferation, Astrocytes, Neurons (↓after CPB)	Ki67, DCX, GFAP. NeuN, SOX2	CPB	Dhari et al. (2021)
	Sheep (Romney)	M	Juvenile (6 weeks)	Proliferation, Differentiation (↔)	BrdU, PCNA, GFAP, NeuN	Exercise (Running)	Swanson et al. (2017)
	Sheep (Romney/Suffolk)	F	Adult young (3 years)	Short/Long-term survival (↔)	BrdU, PCNA	Descriptive	Low et al. (2013)
			Adult old (5–6 years)	Proliferation (↔)			
	Sheep (Île-de-France)	F	Adult (1–2 years)	Evidence for different cell types in SVZ Proliferation (↓)	BrdU, Ki67, DCX, GFAP	Descriptive/Parturition/Lactation	Brus et al. (2010)
	Cattle (n.a.)	F	Adult (1–3 years)	Distribution of ependymal, subependymal cells, astrocytes and proliferating cells	GFAP, PCNA, S100ß, TuJ1/ßIII-tubulin	Descriptive	Rodriguez-Perez et al. (2003)
	Cattle (n.a.)	n.a.	Cell culture (2 years old brains)	Distribution of ependymal, subependymal cells, astrocytes and proliferating cells	BrdU, PCNA, S100ß GFAP, TuJ1/ßIII-tubulin	Descriptive	Perez-Martin et al. (2003)
OB	Sheep (Île-de-France)	F	Adult (1–2 years)	Evidence for different cell types in OB Proliferation (↓)	BrdU, Ki67, DCX, GFAP	Descriptive/Parturition/Lactation	Brus et al. (2010)
	Sheep (Île-de-France)	F	Adult (2–3 years)	Stem progenitor (↓ with time since BrdU) Neuroblasts (↑ 2 and 3 m post BrdU) Neuronal differentiation (↑ with time since BrdU) Glial cells (↑ 2 and 3 m post BrdU)	BrdU, DCX, NeuN, SOX2, S100ß	Parturition (1, 2, 3, 4, and 8 m post BrdU)	Brus et al. (2013)
	Sheep (Île-de-France)	F	Adult (1.5–2 years)	Proliferation (↓ with parturition) Neuroblasts (↑ without lamb; ↓with lamb) Neuronal differentiation (↔ with parturition) Survival (↔)	BrdU, Ki67, DCX, NeuN	Lactation/Interaction with young	Brus et al. (2014)
	Sheep (Île-de-France)	F	Adult (2–3 years)	Neuroblast activation (↑with own lamb exposure) Neuronal activation (↔)	BrdU, Zif268, DCX, NeuN	Lactation/Interaction with young (own or alien lamb)	Corona et al. (2017)
	Sheep (Île-de-France)	F	Adult (4–5 years)	Neuroblasts (↓ by OXT treatment)	DCX	Effect of OXT	Levy et al. (2019)
HYP	Sheep (Île-de-France)	F	Adult (2 years)	Multipotent NSCs in sheep hypothalamus Proliferation (↑during short photoperiod)	BrdU, Ki67, SOX2, NeuN, GFAP, S100ß	Descriptive; Seasonal variations (short vs. long photoperiod)	Migaud et al. (2011)
	Sheep (Île-de-France)	F	Adult (1.5–2 years)	New hypothalamic cells exhibit neuronal and glial phenotypes	DCX, PCNA, SOX2, GFAP	Descriptive	Batailler et al. (2014)
	Sheep (Île-de-France)	F	Adult (1.5–2 years)	Neuroblasts (↑during short photoperiod) Migratory pathway identified Migratory neuroblasts (↑ during short photoperiod)	DCX	Descriptive; Seasonal variations (short vs. long photoperiod)	Batailler et al. (2016)
					Cdk5		
	Sheep (Île-de-France)	F	Adult (4–5 years)	Neuroblasts (↑) Proliferation (↔)	DCX	Effect of OXT	Levy et al. (2019)
					SOX2		

CPB, Cardiopulmonary bypass; d, Days; DG, Dentate Gyrus; F, Female; HIPPO, Hippocampus; M, Male; m, Month; n.a., Not available; OXT, Oxytocin; Ref, Reference; TBI, Traumatic brain injury; w, Weeks.

Although the DG is widely accepted as the neurogenic niche of the hippocampus in the majority of mammalian species investigated, thus sharing regional similarities across livestock animals (Amrein, 2015; Vadodaria and Gage, 2014), there are profound inter-species differences in the organisation and dynamics of hippocampal neurogenesis, affecting timing of generation, migration and differentiation of new neurons. More specifically, whereas in rodents, mature neurons in the GCL are already seen 7–10 days post BrdU injection, with a peak 2 weeks after labelling and a decline 30 days later (Brown et al., 2003; Imayoshi et al., 2008; Kempermann et al., 2003; Petreanu and Alvarez-Buylla, 2002; Steiner et al., 2004; Suh et al., 2007; Winner et al., 2002), hippocampal neurogenesis in sheep is characterised by a slower neuronal maturation compared to non-human primates. Thus, while few (~13%) granule cells express DCX or NeuN 1–3 months following BrdU injection, the proportion of mature neurons reaches a peak level of about 37–50% 4 months after labelling, with a stable amount of surviving neurons observed up to 6–8 months later (Brus et al., 2013; Kohler et al., 2011; Kornack and Rakic, 2001). The fact that there tends to be an increase in BrdU+ cells 4 months after labelling in the GCL, whereas such an increase is seen 8 months post-BrdU in the SGZ, might suggest a migration process of cells from the GCL to the SGZ over time (Brus et al., 2013). Interestingly, around 69% of BrdU+/SOX2+ cells are located in the external layer adjacent to the SGZ rather than in the GCL 1 month post BrdU, suggesting that the SGZ of the DG mostly harbours progenitor and neural stem cells in sheep (Brus et al., 2013).

However, the above results must be interpreted cautiously, given that those studies were performed on post-parturient female sheep. Thus, hormonal changes and/or fluctuations associated with pregnancy/parturition may have modified the observed neuronal maturation.

Unfortunately, no data is available so far on the dynamics of hippocampal neurogenesis in pigs. Although one study on Göttingen minipigs revealed numerous BrdU+ cells along the rostrocaudal and dorsoventral extent of the DG with the hilus as the major site of new granule neuron production in juvenile (6–7 weeks) and adult (32-week-old) pigs (Guidi et al., 2011), it does not provide any information needed to create a picture about dynamics as reported in sheep. This is mainly because most studies that have been performed on pigs (Huguet et al., 2023; Val-Laillet et al., 2017; Val-Laillet et al., 2018; Val-Laillet et al., 2020) use Ki67 as a proliferation marker (see also Table 1). Although Ki67, PCNA and phosphohistone 3 allow conclusions to be drawn about cells that were actively proliferating, they neither provide any information on the fate of specific cells nor on the rate of survival, which can be analysed when using BrdU injections as a birth-dating method. Comparable studies performed on sheep would therefore be desirable to increase our knowledge of the dynamics of hippocampal neurogenesis in other farm animal species. Despite this absence, a study by Franjic et al. did draw a very detailed picture of anatomically defined cell populations in five hippocampal-entorhinal subregions of the adult mouse, pig, macaque and human brain (Franjic et al., 2022). Using single-nucleus transcriptome profiling via snRNA-sequencing, they not only identified highly diverse cell populations with clear regional distinctions across the DG and CA1-CA4 regions of the hippocampus; they also pointed out fundamental species differences in hippocampal DCX expression per cell type and thus delineated the molecular diversity of cytoarchitectural transition from the allocortex to the neocortex. More specifically, they found differing cell-type proportions of DCX+ cells within species, with pigs showing a dominant (55.6%) DCX+ signal within the neural intermediate progenitor-cell niche, suggesting that this cell population may play a specific role in the pig hippocampal system that will need more detailed investigation in future.

Up to now, there generally seems to be a focus on using the immature DCX neuron marker in pig studies. As a result, and because different dietary components and diet enrichments are known modulators of neuroplasticity and cognition, DCX immunolabelling has been used to assess the effect of a high-fat diet +/− omega-3 and *Bifidobacterium breve* enrichment in prepubertal female piglets, revealing an increase in immature neurons after 10 weeks of probiotics and omega-3 fatty acid supplementation (Huguet et al., 2023). Interestingly, the effect was limited to the dorsal hippocampus, which is known for its role in cognition and memory, whereas no effect was observed in the ventral hippocampus, which is more involved in regulating emotions (Jinno, 2016, for review). Similarly, studies by Val-Laillet investigated the effects of different dietary compositions and supplements on hippocampal granule cell layer size, proliferation (Ki67), immature neurons (DCX), working and reference memory in Landrace and Yucatan minipigs (Val-Laillet et al., 2017; Val-Laillet et al., 2018). Meanwhile, the maternal western diet (WD) during gestation led to a shrinkage of the GCL in WD piglets. However, despite an increase in proliferation at 3 months of age, DCX+ cells were demonstrably diminished. Moreover, in spite of the negative effect of WD on immature neuron production, WD piglets performed better on a working and reference memory task, which may suggest that improvements in cognitive abilities are not necessarily linked to alterations in neuroplasticity. It may, instead, reflect a higher motivation in WD piglets to be given the food rewards used in the respective tests (Val-Laillet et al., 2017). In contrast, oral administration of butyrate, known for its positive effect on memory, possibly via upregulation of neurotrophic factors in the context of stress-induced anxiety (Moretti et al., 2012; Valvassori et al., 2014), led to a larger hippocampal GCL volume, accompanied by increased proliferation and DCX+ cell numbers (Val-Laillet et al., 2018). However, these results are hardly comparable because of the difference in breeds and assessment time points used in the experimental outline.

Despite the analysis of immature neuron production in most studies that assess hippocampal neurogenesis in pigs, there has been no use of other neurogenesis markers such as PCNA, SOX2, NeuN and GFAP. Viewing hippocampal neurogenesis as a complex mechanism, where proliferation, differentiation, migration and integration are intricately linked and finely balanced processes, illustrates the need for further studies that provide images of hippocampal neurogenesis in the pig brain. Furthermore, another important factor underestimated in studies assessing hippocampal neurogenesis in farm animals is sex. Indeed, sexual dimorphism in neuroplasticity is a well-known phenomenon in rodents and other mammalian species (Bowers et al., 2010; Hillerer et al., 2013; Zhang et al., 2008 and see Hillerer et al., 2019; Yagi and Galea, 2019; Yagi et al., 2020, for review). Although a study by Conrad et al. using BrdU injections to determine the survival of newly generated cells in the DG tends to suggest comparable sex differences in neonatal pigs with increased levels of cell survival in males compared to females (Conrad et al., 2015), a detailed picture, as in rodents, (Hillerer et al., 2013) is largely missing during different pig developmental stages.

Our understanding of hippocampal neurogenesis in pigs is still at an early stage due to a lack of studies. However, our knowledge of neuroplasticity in the sheep brain, particularly regarding social interaction, gestation, parturition and lactation, is relatively broad. This is mainly due to the group centred around Levy and Brus, who significantly contributed to the research in this field. They revealed a clear effect of the peripartum period on hippocampal neuroplasticity in Île-de-France sheep, comparable to studies in rats and mice, and depicting a distinct clear modulating effect of pregnancy and/or lactation on hippocampal cell proliferation, survival and differentiation levels (Darnaudery et al., 2007; Furuta and Bridges, 2005; Hillerer et al., 2014b; Leuner et al., 2007; Shingo et al., 2003). Using BrdU as a marker of cell division and specific markers of neuroblasts (DCX), primary neuronal progenitors (GFAP) and mature neurons (NeuN), they investigated the effect of parturition and interaction with the young on hippocampal neurogenesis in four experimental groups (Brus et al., 2010; Brus et al., 2014). Moreover, as an additional readout parameter for neuroplasticity, they conducted a morphological analysis of dendritic length and number of nodes of neuroblasts (BrdU+/DCX+) in the DG (Brus et al., 2014), as motherhood is seen to be accompanied by changes in the morphology of new neurons in mice (Kopel et al., 2012). As many as 40–50% of BrdU-labelled cells contained GFAP, suggesting the presence of neural stem cells (Brus et al., 2010). As seen earlier in rats, cell proliferation 24 h post BrdU injection (Brus et al., 2010), cell survival 4 months after BrdU injection, as well as the proportion of new neurons (BrdU+/NeuN+) (Brus et al., 2014) was significantly lower in both parturient groups compared to virgins (Brus et al., 2010; Brus et al., 2014) or a group of ewes interacting with either a male or another female (Brus et al., 2010). In contrast, separating the ewe from the young for 48 h did not affect cell survival or neuronal differentiation (Brus et al., 2014), suggesting that the suppression is more associated with parturition than dependent on any interaction with the young. Given that neither parturition nor separation nor their combination affects dendritic plasticity of neuroblasts in the DG, suggests that other neurogenic zones, such as the OB (see Chapter 3.4), might be predominantly involved in olfactory recognition learning of the lamb.

Exercise is another factor well known for its positive effect on hippocampal neurogenesis in rodent studies and it could be particularly interesting in the context of EE and, thus, livestock welfare. However, only one study so far has addressed the hypothesis that an exercise-induced increase in hippocampal neurogenesis also occurs in larger mammalian brains like those of farm animals. More specifically, Swanson et al. performed a four-week running regimen in 6-week-old male Romney sheep injected with BrdU on 5 successive days, beginning 17 days pre-killing to label proliferating cells in the SGZ (Swanson et al., 2017). However, contrary to the positive effects of exercise on cell proliferation found in rodents (Farmer et al., 2004; Kempermann et al., 1997a; van Praag et al., 1999a; van Praag et al., 1999b; van Praag et al., 2005), this study failed to observe any changes in the number or distribution of BrdU+ cells in sheep SGZ. Furthermore, there was no co-labelling of BrdU with mature neuronal or glial markers (PCNA, NeuN, GFAP) used in this study, suggesting that the neurogenic niche of lissencephalic brains may have a greater proliferative potential than that of gyrencephalic brains, at least in the context of enrichment via exercise. Nevertheless, the positive correlation between the distance covered and the level of cell proliferation in the SGZ (van Praag et al., 1999a; van Praag et al., 1999b) and the fact that the distance covered by sheep in this study was comparably shorter to that in rodent studies, may have affected this outcome. Moreover, the results suggest that other EEs such as odour or auditory components may be better used to positively affect hippocampal neurogenesis in sheep. Indeed, effects on stress response (i.e., reduced CORT levels; Li et al., 2020), neuroplasticity, structure and brain neurochemistry, along with neurotrophic factors related to neuroplasticity, seem to be a common phenomenon of musical interventions in humans, pigs, cattle and chickens (Alladi et al., 2002; Cloutier et al., 2020; de Jonge et al., 2008; Kim et al., 2013 and see Alworth and Buerkle, 2013, for review), when carefully selected within a frequency range of 4,000–16,000 Hz. In this context, BDNF seems to be an important outcome measure, particularly given the fact that increased BDNF levels in the dorsal hippocampus are not only indicative of greater resilience to stress (Mosaferi et al., 2015), but moreover, given its known association with increased levels of neuroplasticity and cognitive function (Novkovic et al., 2015 and see Kraemer and Kraemer, 2023; Liu and Nusslock, 2018, for review). Adding important knowledge about the potential of EE for promoting neuroplasticity in farm animals, a recent study in pigs suggests that auditory EE has a positive effect on BDNF serum levels in piglets of sows exposed to music therapy during the last trimester and/or during lactation (Lippi et al., 2022). In particular, sows have been exposed to classical music (a 2 h playlist chosen at random; sound intensity of 60–75 dB; three times throughout the day) either (1) during the last trimester, but not lactation, (2) through lactation, but not pregnancy, (3) through pregnancy and lactation or, (4) not exposed to music at all. Although music therapy did not change BDNF levels of piglets at birth in any group, exposure to music *in utero* and after birth prevented a decrease in BDNF, as observed in the other groups (Lippi et al., 2022). Although BDNF levels did not affect behaviours associated with stress resilience (i.e., novel arena test, novel-object test, vocalisation, voluntary approach test), it has to be borne in mind that factors like the period of behavioural assessment and the behavioural tests used may play important roles. Moreover, BDNF levels in serum may not necessarily mirror BDNF levels in the hippocampus, which might have affected the study’s outcome.

Thus, there is a need for future studies that (1) assess the effect of EE on other parameters of neuroplasticity, e.g., adult hippocampal neurogenesis, and (2) correlate it with neurotrophin levels (e.g., BDNF) in saliva, serum and locally in the brain, and (3) correlate it with well-validated behavioural outcome measures of anxiety, depression and cognition in pigs.

### 3.3 Neurogenesis in farm animals’ SVZ

Aside from the DG of the hippocampus, the SVZ of the lateral ventricle is a major site of neuroplasticity throughout mammalian species (Altman and Das, 1965; Brazel et al., 2003), including farm animals like pigs (Dhari et al., 2021; Guidi et al., 2011; Kinder et al., 2019; Maeda et al., 2020) sheep (Brus et al., 2010; Low et al., 2013; Migaud et al., 2015) and cattle (Perez-Martin et al., 2003; Rodriguez-Perez et al., 2003). The SVZ is typically divided into four rostrocaudal regions, the anterior, central, lateral and dorsolateral regions (Brazel et al., 2003; Quinones-Hinojosa et al., 2006), with the anterior-dorsolateral (rostral) part being the most active neurogenic niche, displaying a high level of proliferating NSPCs, at least in human and porcine SVZ (Morton et al., 2017; Quinones-Hinojosa et al., 2006). Using BrdU labelling in female juvenile and adult Göttingen minipigs, Guidi et al. revealed numerous BrdU+ cells organised in clusters along the lateral wall and roof of the rostral SVZ. In contrast, only a few were visible in the caudal region of the SVZ (Guidi et al., 2011). Immunohistochemistry studies in seven-week-old pigs revealed a laminar organisation of the anterior-lateral SVZ composed of four distinct layers, sharing structural features of the human SVZ, i.e., an ependymal layer (I) acting as boundary with the lateral ventricle, a layer rich in DCX+ neuroblasts (II), a GFAP+ rich astrocyte ribbon (III), the thickness of which may vary, as well as a transitional zone (IV), rich in myelinated fibres (Morton et al., 2017). SVZ-derived neural stem/progenitor cells proliferate as multipotent neurospheres that have been shown to express the SOX2, GFAP, DCX and Ki67 neural progenitor and neurodevelopmental markers, whereas PCNA labelling as observed in humans is hardly visible in sheep and pig SVZ (Curtis et al., 2005; Morton et al., 2017). DCX+ neuroblasts increase over the neurodevelopmental period in porcine SVZ, with an increased number of clusters seen in prenatal day (PD)42 Yorkshire/Landrace piglets compared to PD16 and PD0 in the anterior end of the SVZ, as well as an increase in labelling on PD16 and PD42 in the anterior portion of the SVZ (Porter et al., 2022). Interestingly, DCX+ neuroblast clusters have been shown to be co-labelled with PSA-NCAM at 2 weeks of age, suggesting a migratory process in the piglet brain (Porter et al., 2022). Indeed, DCX+ cells migrate short distances to the GCL, incorporate and functionally integrate into the hippocampal circuitry (Nakatomi et al., 2002), where they play an important role in hippocampal-dependent memory functions, as well as in long-term potentiation (LTP) that underlies those memory functions (Snyder et al., 2001; Winocur et al., 2006). Besides these migratory processes to the GCL, DCX+ cells with morphologies of migrating neuroblasts have also been found in the striatum and frontal cortices, where they primarily differentiate into calretinin (CalR+) interneurons, suggesting that SVZ is the main source of new neurons in the neocortex (Brazel et al., 2003; Morton et al., 2017). However, cortical development is a dynamic and complex process, and very little is known about the contribution of SVZ to cortical growth and associated cognitive functions in gyrencephalic species, including farm animals (Lewitus et al., 2014; Poluch and Juliano, 2015).

In cattle, neurogenesis in SVZ has been shown and characterised *in vivo* in the bovine lateral ventricle via IHC (Rodriguez-Perez et al., 2003), as well as *in vitro* in explants from the walls of the lateral ventricle (Perez-Martin et al., 2003). Using specific markers such as ßIII-tubulin, S-100ß, vimentin, GFAP and PCNA, Rodriguez-Perez et al. revealed a similar laminar organisation of the bovine ventricular wall, consisting of specific cell types comparable to those seen in pigs. In particular, four main cell types have been identified within the bovine lateral ventricle: ependymal cells, subependymal neuroblasts, astrocytes and PCNA+ proliferating cells (Rodriguez-Perez et al., 2003). Although the latter are found throughout the lateral ventricle, the rostral horn showed highest densities of PCNA+ nuclei within the subependyma that penetrate to the subjacent glial network and migrate in a rostro-ventral direction, thus mirroring the results in pigs and other mammals. Confirming the results of the *in vivo* study, an *in vitro* model using ependymal-subependymal explants from the lateral ventricles of two-year-old calves found a comparable immunoreactivity of GFAP and PCNA in nuclei and basal processes of those cells, further supporting the idea that subependymal cells are the main source of proliferative activity in cattle SVZ (Perez-Martin et al., 2003). Many of the newly generated cells developed neurites and expressed the neuronal marker ßIII-tubulin, which suggests differentiation of cells into neurons (Perez-Martin et al., 2003).

Summarising the results from ependymal and subependymal cells of the SVZ in farm animals, it seems that the organisation of SVZ in distinct layers is similar in pigs, cattle and sheep (Low et al., 2013) and thus comparable to that seen in humans. However, although there is only one study in sheep investigating proliferation dynamics of the SVZ, it is postulated that the rate of maturation of new neurons is much slower in rodents. Generally, it seems that there is an enormous variation in proliferation between species (Curtis et al., 2005; Gould et al., 1999a; Lazic et al., 2006 and Kempermann and Gage, 1998, for review). Whereas PCNA labelling revealed a rate of about 50 cells per mm^2^ in the human SVZ (Curtis et al., 2005; Low et al., 2011; Lucassen et al., 2010, for review), the proliferation rate in mice is around 800 times higher (Gil et al., 2005). Adding information about neurogenesis in the SVZ in rodents and humans, a study in young (3-year-old) and old (5–6-year-old) Romney/Suffolk ewes successfully shed light on proliferation, short (11 days) and long-term (122 days) survival, as well as differentiation at least in one livestock species. Using a combination of BrdU (40 mg/kg; injected over 5 days), PCNA as a proliferation marker, and NeuN as a marker of neuronal fate, Low et al. revealed proliferation counts of approx. 65 BrdU+ cells per mm^2^, thus comparable to proliferation levels in other sheep breeds and experimental setups (Brus et al., 2010). PCNA labelling was much less than in human studies (Curtis et al., 2005) and independent of age (Low et al., 2013). As suggested by previous studies in larger animals, stem cell to neuronal maturation time was significantly slower than in rodents (Eriksson et al., 1998; Kohler et al., 2011; Malberg and Duman, 2003), with only 20% of BrdU+ cells showing a neuronal phenotype (BrdU+/NeuN+) after a survival time of 4 months (Low et al., 2013).

As discussed above for the hippocampus, there is a similar lack of research in pigs assessing the effects of acute/chronic stress and/or EE on neuroplasticity in the SVZ, but moreover, a general lack of studies focussing on porcine SVZ as a neurogenic region in other contexts. Due to its structural similarity to the multi-layered human SVZ (Morton et al., 2017), most studies so far have used the gyrencephalic pig brain (Landrace and Yorkshire pigs) to model the effects of pathological insults such as hypoxic ischaemia (Ara et al., 2010), TBI (Costine et al., 2015; Kinder et al., 2019) or cardiopulmonary pathologies/insults (Dhari et al., 2021; Maeda et al., 2020; Morton et al., 2017) on neuroplasticity and cortical maturation in humans.

Although most of the studies on sheep focus on the OB as a neurogenic region (see below), a few assess the effects of the hormonal environment, for example, seasonal changes and peripartum-associated changes on the ovine SVZ. Thus, administration of BrdU in adult female sheep in July and December, during a long vs. a short photoperiod, revealed no change in SVZ proliferation rate (Migaud et al., 2011), suggesting that changes might be linked and/or limited to structures involved in seasonal neuroendocrine regulation such as the hypothalamus (see also Chapter 3.5). Indeed, endocrine (changes in oestrogen/progesterone levels) and/or social factors (e.g., pregnancy, parturition and interaction with the young) are important regulators of the production of new neurons in the SVZ, not only in rodents but also in sheep (Levy et al., 2017, for review). Injecting 20 mg/kg BrdU 24 h before sacrifice to determine cell proliferation in the ovine SVZ, Brus et al. found parturition as a down-regulatory factor (Brus et al., 2010), as seen in rodents (Darnaudery et al., 2007; Hillerer et al., 2014b; Leuner et al., 2007). Using Ki67 and GFAP as further markers, they also revealed that 40–50% of the 1-day-old BrdU+ cells in the SVZ were GFAP+, suggesting presence of neural stem cells (Brus et al., 2010).

For a summary of the results discussed regarding the SVZ, see also Table 1.

### 3.4 Olfactory bulb neurogenesis in farm animals

Although the ability of the OB to produce new cells is about 10 times lower than that of the SGZ and the SVZ (Gritti et al., 2002, for review), 1-day-old BrdU+ cells co-labelled with Ki67 and GFAP have been observed in the periventricular layer (Chojnacki et al., 2009), suggesting a neurogenic niche within the OB (Brus et al., 2010; Doetsch et al., 1999). Thus, even if the OB may not necessarily be seen as a neurogenic region on its own, there is no doubt that it plays a vital role in neuroplasticity and its associated behaviours. In the olfactory system, neural stem cells function as primary neurogenic and gliogenic precursors in the SVZ located on the wall of the lateral ventricles. They produce transient amplifying cells, which rapidly create chains of neuroblasts that migrate along the RMS to the OB (Brus et al., 2010; Brus et al., 2013; Doetsch et al., 1999; Sawamoto et al., 2011), where the majority of them mature into granular interneurons. Whereas reports about olfactory neuroplasticity are fairly limited or even absent in pigs and cattle, detailed knowledge exists about the dynamics and maturation of new-born cells in the SVZ-OB network in sheep, at least in the context of parturition and lactation. Using BrdU (20 mg/kg) as a marker of cell division and a combination of neuroplasticity markers (SOX2, DCX, NeuN and S100β), Brus et al. have significantly contributed to our understanding of the maturation timeframe of new-born cells in post-parturient sheep OB (Brus et al., 2013). When analysing different BrdU post-administration survival times over eight months (1 m, 2 m, 3 m, 4 m and 8 m), they found no variation in BrdU intensity, except for a decrease at 8 month post-injection, suggesting a slow process of apoptosis over this period. However, the survival time in sheep seems to be much longer than in rodents, where half of the neurons die within the first month after birth (Lemasson et al., 2005). Similarly, while very few neuroblasts (BrdU+/DCX+) were found 1 month after BrdU injection in the granule layer of the OB, a gradual increase was observed in the periventricular layer 1–3 months later, subsequently reaching a plateau and suggesting a far longer maturation time in sheep than in mice, where interneurons become morphologically mature within 4 weeks of birth (Petreanu and Alvarez-Buylla, 2002). Thus, although there is a lack of knowledge about proliferation and/or survival dynamics in pigs, the results in macaques, which reveal a neuronal maturation time of 97 days (Kohler et al., 2011), suggest a comparable neuronal maturation time across gyrencephalic species. Analysis of the other markers used revealed a variation in specific labelling across survival times, with BrdU+/SOX2+ cells (quiescent cells that may start proliferating, depending on physiological changes) decreasing within 1–4 months and a BrdU+/NeuN+ labelling remaining low 1–2 months after BrdU injection and significantly increasing thereafter up to 8 months. Analysis of the glial marker S100 confirmed a significant increase of double-labelled BrdU+/S100+ cells 2–3 months post-injection, with a decline 4–8 months later (Brus et al., 2013).

Aside from providing insights into dynamical neuroplasticity processes within the SVZ-OB network, the group around Brus also elegantly provided evidence for an intricate migratory pathway along the ventricle from the SVZ to the OB (Brus et al., 2013). By using injections of an adeno-associated virus coupled with an enhanced green fluorescent protein (AAV5-eGFP) that infects neural cells in the SVZ, they revealed fusiform cells with and without processes [neuroblasts (20%) and progenitor cells (30%)] in the periventricular and granular layer of the OB one-month post-injection of the virus. Interestingly, eGFP+ cells were only found in the OB when the injection site was located above the SVZ. In contrast, no labelling was present when located too posteriorly on the level of the corpus callosum, further supporting the idea of a neural stem cell lineage within the SVZ.

Despite the knowledge of the mechanisms of neuroplasticity within the SVZ-OB network, the nature and specific contribution of adult-generated neurons to olfactory behaviour is still being debated (Holmes, 2016; Lazarini and Lledo, 2011, for review). However, given that adult-born neurons have unique properties of synaptic plasticity compared to early-born neurons (Nissant et al., 2009), they could indeed provide an important substrate in olfactory learning, which will be discussed in the following section.

It is now well accepted that the peripartum period and the physiological/hormonal changes associated with pregnancy, parturition and lactation modulate neuroplasticity in all the neurogenic sites and species investigated so far (Galea et al., 2014 and Hillerer et al., 2014a, for review). In sheep, where social behaviours, including maternal behaviour, largely rely on an appropriate sense of smell (Corona et al., 2017; Corona et al., 2018; Gelez and Fabre-Nys, 2006; Keller and Levy, 2012), neuroplasticity may provide an additional mechanism that enhances olfactory learning, as well as the establishment of maternal behaviour and a selective bond with the young following parturition (Poindron et al., 2007, for review). Indeed, a single BrdU treatment (20 mg/kg) 24 h before sacrifice and additional Ki67 labelling revealed a parturition-dependent down-regulation of cell proliferation in the SGZ, SVZ and OB in ewes (Brus et al., 2010), which may be a mandatory mechanism for facilitating the olfactory perceptual memory demands associated with maternal behaviour by favouring the survival and integration of neurons born earlier. Furthermore, co-labelling of the one-day-old BrdU+ cells with GFAP showed that 40–50% of those cells were positive for the respective marker, suggesting the presence of neural stem cells in the SVZ and OB of the ewes (Brus et al., 2010). The density of these BrdU+/GFAP+ cells was around three times higher around the ventricle than the granular and periglomerular cell layer, which further supports the hypothesis of a neurogenic zone in the ventricular margin of the OB.

Interestingly, as previously observed in rodents (Leuner et al., 2007), one major important regulator of peripartum-associated changes in neuroplasticity in sheep seems to be the presence of and particularly interaction with the young. More specifically, Brus et al. used three different groups of sheep in their experimental outline. A virgin control group, a group of ewes allowed to interact with their young 2 days after parturition and a group of ewes that had their lambs removed 2 days before sacrifice (Brus et al., 2014). Immunohistochemical analysis uncovered a suppression of cell proliferation and survival in the OB, which was dependent on lamb presence. Interestingly and contrastingly, in the SGZ, suppression was more likely to be associated with parturition. It is hypothesised that fewer neurons reduce cell competition in the OB, thus enhancing the maturation of new neurons selected to participate in odour learning by young animals. However, olfactory learning seems to involve other mechanisms of neuroplasticity apart from changes in cell proliferation and survival. Thus, neurochemical and electrophysiological changes in mitral cells of the OB supporting the coding of the lamb were shown more than 30 years ago (Kendrick et al., 1992; Keverne et al., 1993; Levy et al., 1993; Levy et al., 1995a). Furthermore, morphological changes at the neuroblast level (DCX+) (for example, dendritic length and number of nodes) are known to be involved in enhancing olfactory neuroblast maturation by lamb interaction (Brus et al., 2014). Indeed, learning has been associated with an accelerated maturation of dendritic trees of new-born neurons in other contexts than lactation (Lemaire et al., 2012; Tronel et al., 2010) and motherhood is a prominent modulator of new-born neuron morphology in rodents (Kopel et al., 2012). Hence, it appears that the ability to process sensory information depends on the functional architecture, synaptic connectivity and electrophysiological changes within the OB. However, adult neurogenesis may be an important regulator in fine-tuning these processes.

Performing two other studies involving Île-de-France ewes, Corona et al. (2017) added further information on the importance of neuroplasticity in the SVZ-OB network in the context of motherhood, olfactory processing, odour learning and maternal selectivity in sheep. Their main questions were: (1) Do adult-generated neurons in the OB contribute to the processing of odours involved in attracting lambs and memorising their individual olfactory signatures? (2) Do olfactory stimuli coming from the neonates specifically activate new interneurons in the OB (Corona et al., 2017)? (3) Does the chemical disruption of olfactory neurogenesis impair the establishment of maternal behaviour in sheep and alter their ability to learn their own lambs’ olfactory signature (Corona et al., 2018) ?. In an attempt to answer the first set of questions, sheep were given four BrdU injections (20 mg/kg) 3 months before parturition. After birth, ewes from Group 1 spent 2 days with their lambs, were then separated for 3 h and re-exposed to either their own, to an unfamiliar lamb or an adult conspecific. In contrast, ewes from Group 2 were not exposed to any lamb for 5 h (control group). Using a combination of BrdU, DCX, NeuN (neuroplasticity markers), as well as c-Fos and Zif268 (neuronal activation markers), they showed preferential activation of three-month old OB neuroblasts (BrdU+/DCX+/Zif268+), but not mature neurons (BrdU+/NeuN+/Zif268+), mirroring the results in rodents where immature olfactory neurons are very susceptible to sensory experience compared to mature neurons (Belnoue et al., 2011; Mouret et al., 2008; Yamaguchi and Mori, 2005). Furthermore, this activation was specific to the OB, as no activation was observed in the SGZ (Corona et al., 2017). Interestingly, neither neuroblasts nor mature neurons of the OB differentiate between exposure to familiar or unfamiliar lambs, suggesting either that older neurons (>3 months) may be involved in recognising their own lambs or that adult olfactory neurogenesis is rather important for the long-term olfactory memory as seen in father mice who exhibited preferential activation of olfactory adult-born neurons when exposed to the odour of 6-week-old offspring (Mak and Weiss, 2010). Nevertheless, the responsiveness of adult-born neurons is lamb-odour specific and not an activation by odours *per se*, as no activation was observed when exposed to an unfamiliar ewe (Corona et al., 2017). To further prove that olfactory neurogenesis is indeed involved in the establishment of maternal behaviour and olfactory signature learning, they administered the mitotic blocker cytosine arabinoside (Ara-C) into the SVZ. Then they assessed maternal bleats and olfactory neurogenesis in the context of lamb discrimination behaviour (Corona et al., 2018). Adding information from previous studies, where they showed a reduction in maternal behaviour rendering ewes anosmic after a zinc sulphate infusion (Levy et al., 1995b), or removing the amniotic fluid from neonates (Levy and Poindro, 1987), Ara-C treatment led to (1) a 70% reduction in olfactory neurogenesis (DCX+ cells) and (2) fewer maternal bleats indicative of maternal responsiveness (Dwyer et al., 1998) during the first hours after parturition and (3) a lack of familiar/unfamiliar lamb discrimination (Corona et al., 2018).

Given the known interplay between the OXT system and the sensory system (Grinevich and Stoop, 2018, for review), the fact that OXT plays an important role in the orchestration of socio-emotional behaviours and the physiological and neuronal maternal adaptations during the peripartum period, another study in non-pregnant sheep investigated the potential involvement of central OXT in regulating adult neurogenesis in the OB (Levy et al., 2019). Ewes received steroid treatment followed by intracerebroventricular infusions of OXT to mimic endocrine events during parturition. OXT significantly decreased the density of the neuroblasts (DCX+) in the OB. In contrast, there was no effect in any other neurogenic niche, such as the SGZ or ventromedial nucleus of the hypothalamus (Levy et al., 2019), although the latter might be due to a lack of OXT-R usually expressed in neuroblasts or more mature neurons, as seen before in rodents and sheep (Broad et al., 1999; Lin et al., 2017). Aside from the region-specific effect of OXT, the effect was also cell-type specific, as the density of progenitor cells was not influenced by OXT treatment (Levy et al., 2019). Consequently, other neuro-modulatory factors such as glucocorticoids could be involved (Sandi, 1998, for review); it seems that OXT participates in the formation of olfactory memory in sheep after parturition by decreasing the density of adult-generated neuroblasts in the SVZ-OB network.

In summary, neural plasticity within the SVZ-OB network during the peripartum period appears to be an adaptive response to the high olfactory perceptual demand at that point, with adult-born neurons helping to process infantile odours.

For a summary of the results discussed regarding the OB, see also Table 1.

### 3.5 Neurogenesis in the hypothalamus of farm animals

The hypothalamus, known as a key regulator of homeostatic activity and numerous physiological functions such as reproduction, food intake and energy balance ((Schneeberger et al., 2014) for review), has not classically been considered a neurogenic niche. However, more than 20 years ago, proliferative activity (BrdU labelling) within the hypothalamus was reported in rats (Pencea et al., 2001b). To date, constitutive hypothalamic neurogenesis has been proven to occur in many species of mammals including mice (Kokoeva et al., 2007; Lee and Blackshaw, 2012; Li et al., 2012; Werner et al., 2012), rats (Pencea et al., 2001b; Perez-Martin et al., 2010; Xu et al., 2005), voles (Fowler et al., 2005), hamsters (Huang et al., 1998) and sheep (Batailler et al., 2016; Batailler et al., 2014; Migaud et al., 2011; Migaud et al., 2010) (for review see Lee and Blackshaw, 2012 and Migaud et al., 2010). Several reports indicate that hypothalamic stem cells are located in the hypothalamic parenchyma (Li et al., 2012; McNay et al., 2012). However, lineage-tracing studies revealed that a subpopulation of tanycytes lining the ependymal layer of the third ventricle (Xu et al., 2005) and expressing the NSC markers SOX2, GFAP, vimentin and nestin (Batailler et al., 2014; Lee and Blackshaw, 2012; Li et al., 2012) are instead neural stem cells with a proliferative capacity within the hypothalamus. From the subependymal niche, they spread towards the neighbouring nuclei [i.e., arcuate (Arc) and ventromedial nucleus (VMH), median eminence], forming a migratory path of progenitors. Those cells, expressing DCX, show a round morphology close to the third ventricle, whereas they are rather fusiform and bipolar deeper in the parenchyma, further suggesting maturation in, or migration through the parenchyma (Batailler et al., 2014; Lee and Blackshaw, 2012). Most of these DCX+ cells will adopt a neuronal phenotype (BrdU+ NeuN+) 1 month after labelling (Batailler et al., 2014; Haan et al., 2013; Migaud et al., 2015). However, the hypothalamus also shows gliogenic and microglial-like properties, at least in sheep, where approx. 70% of new cells differentiate into S100ß and GFAP+ astrocytes (Steiner et al., 2004) and 10% CD45+ cells, respectively, (Hazlerigg et al., 2013), with no evidence for oligodendrocytic labelling (Steiner et al., 2004).

Naturally enough, the obvious proliferative capacity of the hypothalamus discussed above raises the question of its functional relevance. The discovery that endogenous and exogenous factors, including the photoperiod and season, may modulate hypothalamic cell proliferation and neuron production (Migaud et al., 2011; Migaud et al., 2010; Migaud et al., 2015) soon suggested the involvement of hypothalamic neuroplastic changes in seasonal cycles of physiology and behaviour, which are essential adaptations to cope with environmental variations and to fine-tune the timing of reproductive/metabolic functions. Indeed, photoperiod effects on proliferative capacity and marked structural changes within the hypothalamus have first been observed in songbirds (Alvarez-Buylla et al., 1990; Goldman and Nottebohm, 1983). Using ^3^H-thymidine to identify dividing cells revealed that new neurons are generated and integrated into functional avian song circuits in a photoperiodic manner (Paton and Nottebohm, 1984). To date, photoperiod-dependent changes in hypothalamic neuroplasticity have also been extensively studied in sheep, a species where sexual activity/inactivity largely relies on photoperiodic length. More specifically, measuring the expression of DCX+ cells at various times of the year, Batailler et al. showed that the density of these cells varied across the photoperiod, with higher counts during the short photoperiod in the Arc and higher counts during the long photoperiod in the VMH (Batailler et al., 2016). Thus, it appears that higher cell proliferation levels are associated with a short stimulatory photoperiod (sexual activity, for example). In contrast, lower proliferation is more likely to be observed during phases of sexual inactivity (i.e., inhibitory long photoperiods). Although peripheral oestradiol levels have been shown to be independent of seasonal changes in cell proliferation in sheep (Migaud et al., 2011), the fact that hypothalamic neurons express ERα (Batailler et al., 2014) and the well-known potential of oestrogens to regulate neuroplastic changes in rats (Cameron and Gould, 1994; Ormerod and Galea, 2001; Tanapat et al., 1999), further suggest an involvement of hypothalamic neuroplasticity in reproductive behaviour and seasonal variation in neuroendocrine secretion. Furthermore, other hormones could be involved in the regulation of hypothalamic neuroplasticity. A study by Levy et al. revealed that intracerebroventricular injections of OXT in non-pregnant female sheep significantly increased the density of neuroblasts in the VMH, with no change in any other neurogenic niche being investigated (i.e., OB and SGZ) (Levy et al., 2019).

In summary, there is some initial evidence for hypothalamic neuroplasticity’s involvement in regulating seasonal changes in neuroendocrine function. Nevertheless, more studies are needed to investigate whether these alterations are also sensitive to EE and how this may relate to animal welfare in livestock farming.

For a summary of the results discussed regarding the hypothalamus, see also Table 1.

## 4 The microbiota-gut-brain-axis and adult neurogenesis in farm animals: associations and future directions to improve behaviour and welfare in livestock farming

**Figure 3:**
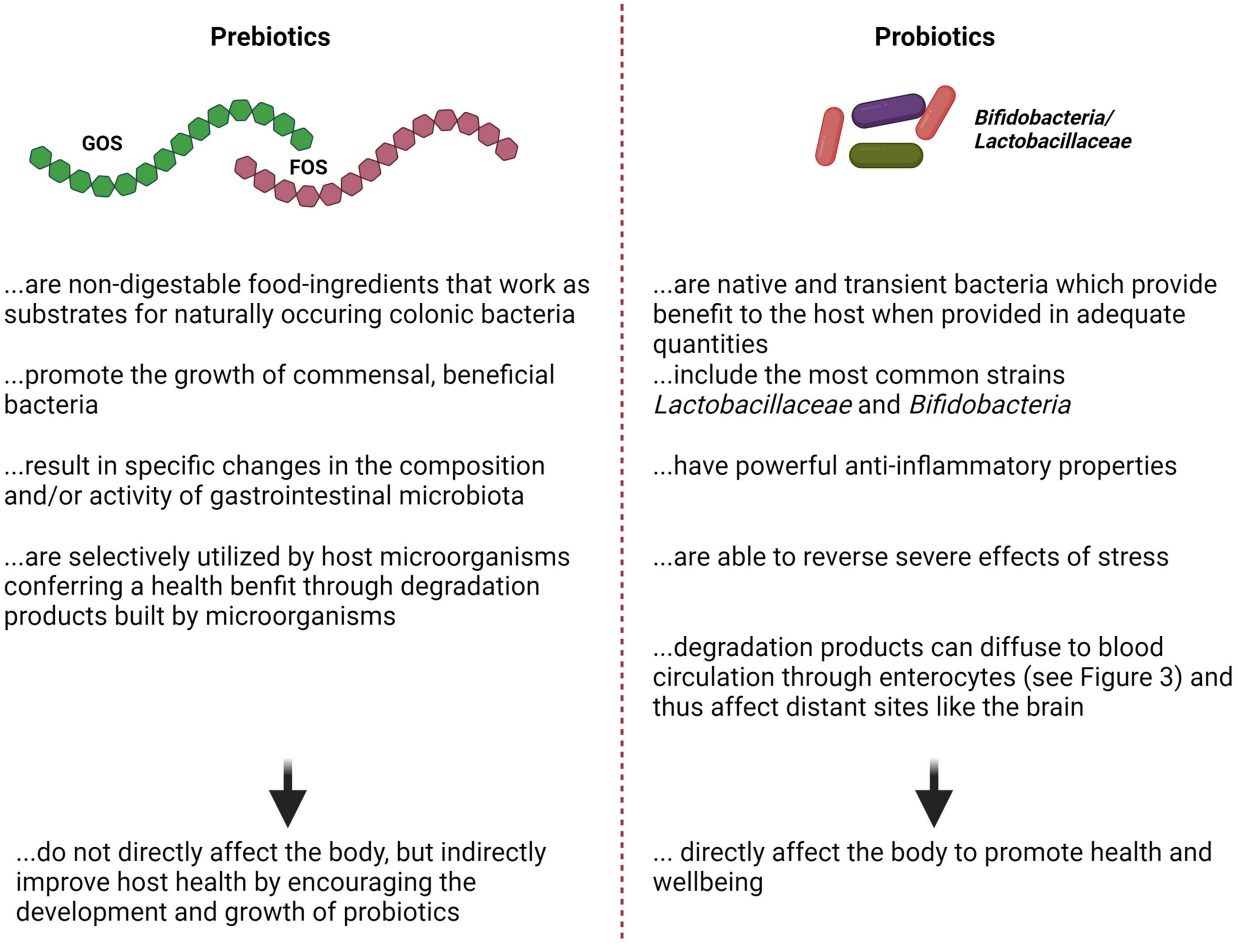
Opposing listing about the features of Pre-vs. Probiotics. References Prebiotics: see (Davani-Davari et al., 2019) for review; References Probiotics: (Isolauri et al., 2002; Laiho et al., 2002) for review. FOS, Fructo-Oligosaccharide; GOS, Glyco-Oligosaccharide.

As discussed in Chapter 2, one possibility for providing EE is to implement nutritional enrichment via diet composition, including pre-or probiotics (for features of pre vs. probiotics, see also Figure 3). Various studies in pigs have shown multiple beneficial effects of probiotic-enriched sow diet on the overall performance (e.g., increased piglet weight, quantity and quality of colostrum and feed consumption during lactation), particularly during sensitive periods such as pregnancy, lactation and early development (Innamma et al., 2023; Kritas et al., 2015; Pereira et al., 2024). Furthermore, the modulation of intestinal microbiota via probiotic treatment is known to have wide-ranging effects on different aspects of farm animal behaviour (Budino et al., 2014; Sapkota et al., 2016) and might thus offer a potentially promising strategy for mitigating aversive effects on animal welfare caused by intensive farming practises.

Unfortunately, knowledge about the targeted use of pre/probiotics in the context of stress, stress resilience and welfare in farm animals is insufficient (Kraimi et al., 2019, for review), but importantly, there is considerable lack of knowledge about its effects on neuronal parameters, particularly neuroplasticity, as well as its association with anxiety or depression-like behaviour and welfare of domestic animals. This is surprising in more than one way: (1) given the literature in rodents, which shows that detrimental effects of stress can be significantly reduced with EE (Bondolfi et al., 2004; Kempermann et al., 1997a; Lazic et al., 2006; Rao et al., 2006); (2) considering the rising amount of studies clearly revealing an association between microbiota supplementation, stress, neuronal parameters and behaviour (Cryan et al., 2019; Guzzetta et al., 2022; Kraimi et al., 2019; Ratsika et al., 2021; Sharvin et al., 2023, for review).

To date, most of our knowledge about the association between EE, stress, microbiota, the gut-brain axis (GBA), and neuroplasticity derives from rodent studies.

The following chapter will first give a brief and general overview of the concept of microbiota and the MGBA, summarise available literature about microbiota manipulation in farm animals, and finally try to draw an outlook about a potential use of pre/probiotics to positively influence neuroplasticity and thus welfare and stress resilience in farm animals.

While it has been known since the 1800s that the gut and the brain communicate with each other to maintain homeostasis, it has become more and more obvious during the last few years that the gastrointestinal tract harbours up to 10^4^ saprophytic microorganisms, including bacteria, viruses, fungi, yeast, protozoans and archaea (Montanari et al., 2023; Rooks and Garrett, 2016, for review) that encompass the gut microbiota. This myriad of symbiotic microbes functions as a ‘second brain’ (Carabotti et al., 2015, for review), which is in bidirectional communication with the brain itself (Luqman et al., 2024, for review). Through the GBA, it has an enormous impact on brain processes (Arzani et al., 2020, for review), exploiting several anatomic structures, systems and metabolic routes such as the HPA axis, the neuroimmune-and the autonomous nervous system, for example (Rao et al., 2006). Thus, the microbiota’s ability to affect the CNS bidirectionally through the parasympathetic nervous system is mainly mediated by neurological autonomous, hormonal (HPA) and immunological (cytokine and chemokine) processes, which are all interrelated (Carabotti et al., 2015; Kennedy et al., 2014, for review). It is now obvious that the microbiota composition has a major influence on neurodevelopment, several aspects of brain function, stress response and, moreover, stress sensitivity (Beurel and Nemeroff, 2024; Borre et al., 2014; Foster et al., 2017; Morys et al., 2024, for review). Knowledge mainly arises from studies in rodents manipulating gut microbiota via antibiotic treatment, application of probiotics, microbiota transfer, or germ-free animals. The latter has been shown to exhibit an exaggerated HPA-axis response to acute stress, which could be reversed by specific *Bifidobacteria* species. Moreover, intestinal microbes are required for normal brain development (Diaz Heijtz et al., 2011) and brain function in adulthood (Stilling et al., 2015). Although studies of the gut microbiome and the MGBA in farm animals are limited for methodological reasons (e.g., housing germ-free animals would require space-consuming isolators), interventions with probiotics reinforce the theory of microbiota’s influence on behavioural and cognitive abilities with an impact on well-being (for review, see Kraimi et al., 2019). More specifically, modification of microbiota activity by maternal diet leads to an increased working memory in piglets (Val-Laillet et al., 2017). Indeed, a recent study in pigs supports the idea that manipulation of the gut microbiota may be transgenerational and that an application during pregnancy may prevent adverse behavioural outcomes in the offspring. Consequently, piglets of sows fed with a multi-strain probiotic during pregnancy until weaning had lower plasma CORT, higher 5-HT levels and lower scores indicative of social fear and aggressive behaviour towards humans (Pereira et al., 2024). Although it is well known that changes in anxiety, social fear and cognition are associated with such key neuronal processes as neurogenesis (Ogbonnaya et al., 2015), myelination (Hoban et al., 2016), BBB integrity (Braniste et al., 2014) and microglial activation (Thion et al., 2018), there is a lack of studies, particularly in farm animals, investigating the influence of gut microbiota or MGBA on neuronal parameters, behaviour, well-being and vice versa.

**Figure 4:**
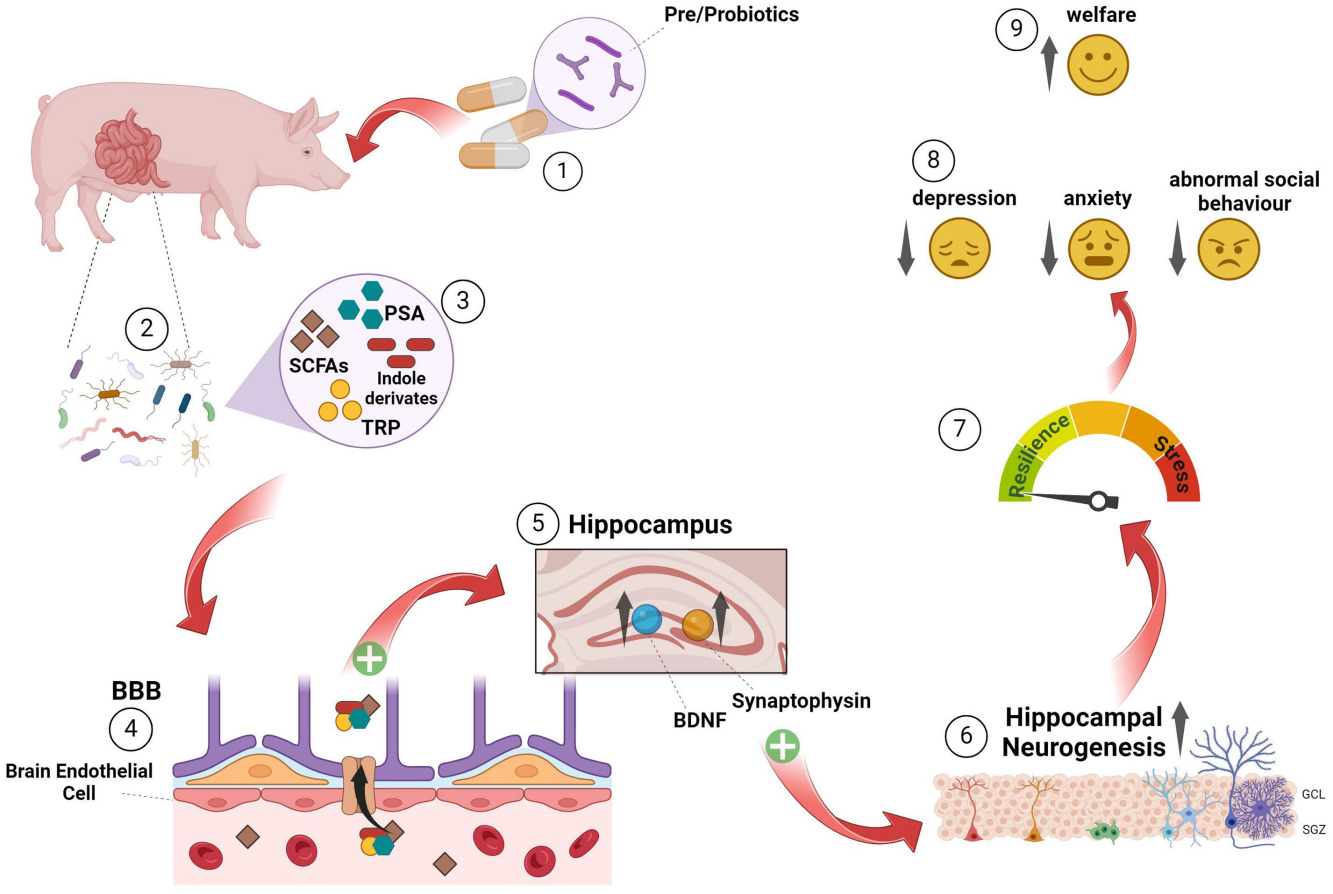
Hypothesised action and association between microbial EE via probiotics, microbiota, adult neurogenesis and welfare in farm animals ① Oral application of probiotics will lead to a colonisation of beneficial microbiota in the gastrointestinal tracts of farm animals; ② metabolites of those microorganisms (SCFAs, PSA, indole derivates and TRP, for example); ③ can diffuse into the blood circulation, crossing the BBB via enterocytes ④; reaching brain regions like the hippocampus, known for their involvement in mood regulation ⑤. Here, they enhance the BDNF and synaptophysin levels, promoting increased hippocampal neurogenesis ⑥. Augmented hippocampal neurogenesis rates are associated with stress resilience ⑦, which will promote a reduction in depressive and anxiety-like behaviours, as well as abnormal social behaviours ⑧ and thus an improvement in the welfare of livestock animals ⑨. BBB, Blood brain barrier; BDNF, Brain-derived neurotrophic factor; GCL, Granular cell layer; PSA, Polysaccharide A; SCFAs, Short chain fatty acids; SGZ, Subgranular zone; TRP, Tryptophan.

However, there is a growing body of evidence from rodent studies that microbiota composition is essential in the context of stress regulation and stress resilience, particularly during vulnerable developmental periods. Furthermore, it might be a key regulatory component of imbalances in neural stem cell proliferative and differentiation activities in brain neurogenic niches, including adult neurogenesis, which have been associated with brain disturbances such as depression, neurodevelopmental and cognitive disorders (for review see Cussotto et al., 2018, Foster et al., 2017, Guzzetta et al., 2022, and Kentner et al., 2019). Therefore, microbiota composition may not only represent a causative determinant but moreover a therapeutic target in the context of behavioural disorders and diseases where adult neurogenesis plays a role (Ribeiro et al., 2020 and see Cerdo et al., 2020 for review). Microbiota modulators involved in the microbiota-GBA-neurogenesis circuit may be intrinsic, e.g., ageing, oxidative stress and inflammation, or extrinsic, such as antioxidants and anti-inflammatory components, polysaturated fatty acids, as well as pre/probiotics (for more information about pre/probiotics see Figure 3). The latter may be of particular interest, as there is growing evidence that targeted manipulations of microbiota by adding pre/probiotics might confer protection to the brain to ameliorate the negative effects of stress due to HPA-axis activation, particularly during vulnerable developmental periods (Sarkar et al., 2016, for review). Although there are no clear connections between pre/probiotics, microbiota and adult neurogenesis, or specific molecular mechanisms underlying the influence of gut microbiota on adult neurogenesis or these connections are not fully understood, the evidence does point to a pivotal role of pre/probiotics, intestinal microbiota and bacteria-derived metabolites in the GBA communication and, in particular, in modulating adult neurogenesis. This is not surprising, given that metabolites released by intestinal microbes may enter the bloodstream, cross the BBB and reach the brain, where they can affect outcome variables (BDNF and synaptophysin, for example) linked with alterations in adult neurogenesis (see also Figure 4). In more detail, the application of *Lactobacillus rhamnosus* in male rats was able to reverse the effects of early-life stress on anxiety in an open field test and on learning in the Morris water maze (McVey Neufeld et al., 2019). Similarly, *Bifidobacterium longum R0175* treatment prevented stress-reactivity and stress-induced changes in neurogenesis in female rats (Ait-Belgnaoui et al., 2012). Furthermore, microbiota-induced reductions in anxiety and improvements in cognition have been shown in germ-free mice, rats and humans treated with *Lactobacillus helveticus* or *Bifidobacterium longum R00052* (Liu et al., 2016; Messaoudi et al., 2011). Pointing out the importance of microbiota during sensitive windows of brain development, a double-blinded placebo-controlled study in humans applying *Lactobacillus rhamnosus* during pregnancy and lactation significantly reduced postpartum anxiety and depression (Slykerman et al., 2017). Although the mechanisms behind these changes are not fully understood, it is suspected that the above-mentioned behavioural changes associated with adult neurogenesis are regulated by an increase in BDNF and synaptophysin mRNA expression in the hippocampus (Corpuz et al., 2018; Distrutti et al., 2014) (see also Figure 4 for graphical presentation).

## 5 Discussion

Exploring the foundations of the neurobiology of farm animal behaviour is essential to better understand how well-being occurs in the brain and why each individual expresses positive or negative emotions. Since all behaviours result from fine-tuned activities in the brain, a better understanding of those processes is the basis for a neurobiological approach that might offer new opportunities to increase welfare in livestock farming.

Although there has been an effort to explore neuroplasticity in farm animals, we are still far behind the basic research on other species. This is mainly due to the comparative infancy of the application of neurobiological techniques within the field of animal welfare research and the difficulty in analysing brain function in livestock species larger than rodents. As environmental effects may differentially shape farm animal behaviour and changing or challenging environments may involve resetting the neuronal, endocrine and immune mechanisms, there is a vital need for future studies not only to assess the fundamental dynamics of neuroplasticity but also to analyse the potential correlations between EE and neuroplasticity in farm animals to improve welfare in livestock farming individually.

The literature suggests that applying pre/probiotics, particularly during sensitive developmental windows, might be an effective EE strategy in animal husbandry systems with the potential to reduce the stress effects of modern animal housing and thus improve farm animal welfare. However, before microbial enrichment can be used to change nutrition and management processes to increase welfare in livestock farming, there is a crucial need for studies assessing the possible effects and relationships of probiotics and the MGBA on neurogenesis in farm animals and how these changes might be associated with behavioural welfare outcome variables like anxiety-, depression-like and social behaviour.
